# Cardiooncology—dealing with modern drug treatment, long-term complications, and cancer survivorship

**DOI:** 10.1007/s10585-021-10106-x

**Published:** 2021-06-12

**Authors:** Claudia de Wall, Johann Bauersachs, Dominik Berliner

**Affiliations:** Dept. of Cardiology and Angiology, Hannover Medical School, Carl-Neuberg-Str. 1, 30625 Hannover, Germany

**Keywords:** Cardio-oncology, Cardiotoxicity, Myocardial dysfunction, Cancer survivorship, Long-term complications, Chemotherapy

## Abstract

Modern treatment strategies have improved prognosis and survival of patients with malignant diseases. The key components of tumor treatment are conventional chemotherapy, radiotherapy, targeted therapies, and immunotherapy. Cardiovascular side-effects may occur in the early phase of tumor therapy or even decades later. Therefore, knowledge and awareness of acute and long-lasting cardiac side effects of anti-cancer therapies are essential. Cardiotoxicity impairs quality of life and overall survival. The new cardiologic subspecialty ‘cardio-oncology’ deals with the different cardiovascular problems arising from tumor treatment and the relationship between cancer and heart diseases. Early detection and treatment of cardiotoxicity is of crucial importance. A detailed cardiac assessment of patients prior to administration of cardiotoxic agents, during and after treatment should be performed in all patients. The current review focusses on acute and long-term cardiotoxic side effects of classical cytotoxic and selected modern drug treatments such as immune checkpoint inhibitors and discusses strategies for the diagnosis of treatment-related adverse cardiovascular effects in cancer patients.

## Introduction

Advances in the early detection of malignant diseases and modern treatment strategies have improved prognosis and survival of cancer patients [1]. Cardiovascular morbidity and mortality is increased in those patients due to pathophysiological processes in connection with the cancer itself, patient initial comorbidities, and toxicity of anticancer therapies making cardiovascular diseases in those patients a leading cause of death after cancer itself [2–4]. As patients with malignant diseases have been largely excluded from randomized controlled clinical trials in cardiology during the past decades evidence on diagnostics, treatment and monitoring strategies is scarce [5]. In contrast, respective treatment strategies based on multiple randomized trials exist in non-cancer patients, emphasizing the need for further cardiovascular research in cancer patients and justifying the relevance of cardio-oncology. The development of new oncological therapeutic agents that specifically influence the central signaling pathways of tumor cells has led to the approval of many new drugs in recent years. In parallel, there are also several reports of cardiovascular side effects. Therefore, the interaction between cardiology and oncology is becoming increasingly important.

Cancer related cardiovascular complications may manifest acutely within days, weeks, or months but also chronically years after the initial treatment. Due to a better precision of modern treatment strategies, physicians are confronted with an increasing number of adult survivors of childhood, adolescent and young adult cancer. Long-term toxicity in this patient group has been reported to be up to 60–70% [6]. Well-structured cancer survivorship programs are of crucial importance to continuously monitor patients with increased life-time risk for morbidity and mortality.

In the last years cardiooncology has emerged as a new field in cardiology targeting on more evidence in the pathophysiology, diagnostics, and treatment of acute and long-term cardiotoxic effects of (modern) cancer treatment strategies.

Online databases (MEDLINE, PubMed) were searched for recent studies and reviews evaluating the cardiac side effects in cancer patients. The following keywords were used: cardiotoxicity, chemotherapy, immune check point inhibitors, radiation therapy, cancer survivors, myocardial dysfunction. Potentially relevant citations were retrieved from reference lists of the identified reports and relevant reviews. We consider the cardiac side effects of conventional cytotoxic chemotherapy, antibodies targeting immune checkpoints, and radiotherapy. In the current review molecular therapies targeting signal transduction are not discussed.

### Cardiovascular side effects of clinically selected anti-cancer therapies

**Fig. 1:**
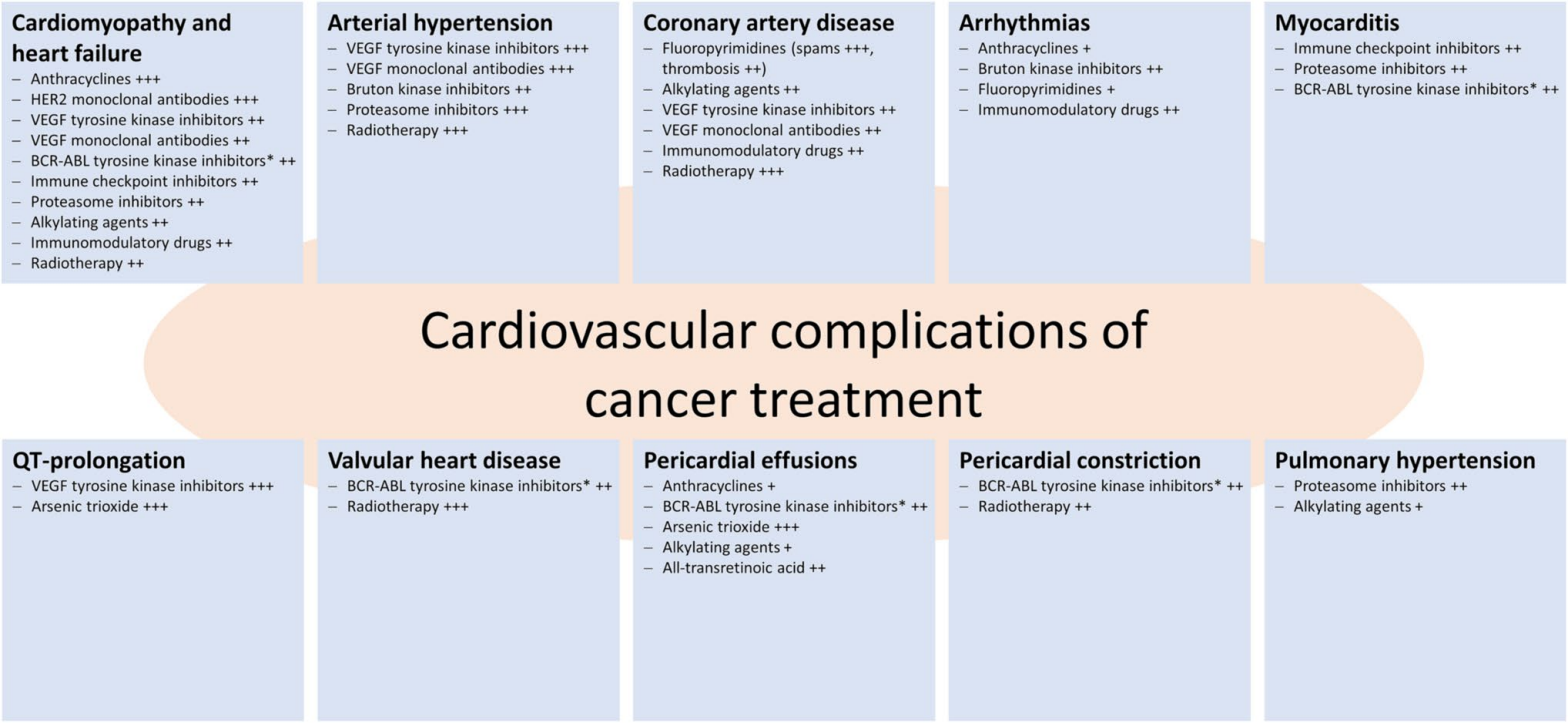
Cardiovascular complications of modern cancer treatment and the associated risk (* drug-dependent risk; + treatment associated with < 1% to develop the form of cardiotoxic side effect, ++ risk estimated to be between 1 and 10%, +++ risk estimated to be > 10%; adapted from [64,86]). For further information, the consensus paper of the European Society for Medical Oncology (ESMO) on “Management of cardiac disease in cancer patients throughout oncological treatment” gives a comprehensive overview of the adverse cardiovascular side effects of the different drug classes as well as their different, specific agents while referring to respective key data and publications (Supplementary Table S1; [86])

The clinical spectrum of cardiovascular complications is broad and ranges from heart failure and cardiomyopathy over valve diseases, myocardial ischemia, pericardial diseases, and hypertension to arrhythmias and thromboembolic complications (Fig. 1). Cardiomyopathy and heart failure are the most common and often most limiting manifestations [7]. Actually, there is no single and universally accepted definition of cancer treatment related cardiotoxicity [5]. Several medical societies have introduced different definitions of cardiotoxicity (Table 1) [8–10].

**Table 1:** Different definitions of cardiotoxicity across organizations

Area of expertise	Medical Society	Region	Definition of cardiotoxicity
Cardiology	DGK	Germany	Asymptomatic or symptomatic LVEF fall by > 10% from baseline to LVEF < 50%
	ESC	Europe	Asymptomatic or symptomatic LVEF fall by > 10% from baseline to LVEF < 50%
	CCS	Canada	LVEF fall by > 10% from baseline or LVEF < 53%
Cardiology—Imaging	EACVI	Europe	LVEF fall by > 10% to absolute LVEF < 53%
	ASE	United States	LVEF fall by > 10% to absolute LVEF < 53%
Oncology	ESMO	Europe	Symptomatic decline in LVEF of at least 5% to < 55% or asymptomatic decline in LVEF of at least 10% to < 55%
	NCI	United States	Common Terminology Criteria for Adverse Events: Heart Failure grade 1-5*

*ASE* American Society of Echocardiography, *CCS* Canadian Cardiovascular Society, *DGK *Deutsche Gesellschaft für Kardiologie (German Society of Cardiology), *EACVI *European Association of Cardiovascular Imaging, *ESC *European Society of Cardiology, *ESMO *European Society of Medical Oncology, *LVEF* left ventricuar ejection fraction, *NCI *National cancer Institute; *Grade 1 (asymptomatic), Grade 2 (mild to moderate symptoms), Grade 3 (symptomatic on minimal exertion or at rest), Grade 4 (life-threatening), Grade 5 (death); adapted from [5, 11, 14, 76, 84, 85]

In 2014, the European Association of Cardiovascular Imaging and the American Society of Echocardiography presented a concordant consensus defining cancer therapeutics-related cardiac dysfunction (CTRCD) as a decrease in the left ventricular ejection fraction (LVEF) of > 10 percentage points to the value < 53% (normal reference value of two-dimensional echocardiography). This decrease should be confirmed by repeated cardiac imaging 2 to 3 weeks later [11].

Based on the underlying pathophysiological mechanism, CTRCD has been classified into two types [11]:

1. Type I CTRCD is caused primarily by *anthracyclines* and leads to cell apoptosis and therefore to an irreversible myocardial cell damage. The effect is dose dependent.
2. Type 2 CTRCD is caused by several different agents (e.g. *trastuzumab*), is not dose dependent, does not lead to apoptosis by itself and therefore may be reversible.

It has to be mentioned that this strict classification in type 1 and type 2 CTRCD is more historic and no longer followed since overlapping outcomes are reported for the two chemotherapeutic classes.

The timing of cardiovascular side effects can vary among agents. In anthracyclines, the damage occurs immediately after exposure [12]. Data from a trial in which 2625 patients with anthracycline-based chemotherapy were enrolled showed an occurrence of cardiotoxicity within the first 12 months after treatment in 98% of patients [13]. In contrast, cyclophosphamide-associated heart failure which is dose-dependent develops typically within 7 to 10 days after the application of high dose regimens [14]. Coronary artery vasospasm caused by fluoropyrimidines (5-FU) also occurs in the early phase of treatment. Cardiac events are typically short-lasting (up to 48 h) and tend to manifest within 2 to 5 days after initiation of 5-FU [15]. Two studies that assessed the timing of cardiotoxicity in trastuzumab treated patients suggest that the first 3 months of treatment are the most precarious ones, counting for most of the cardiotoxic events, and that cardiotoxicity occurring more than 6 months after start of trastuzumab is rare [16, 17]. Similarly, most cases of ICI-induced myocarditis occur in the first three months following the start of treatment [18]. In this context, a special group of patients should be emphasized: There is evidence that pregnancy subsequent to childhood malignancy and chemotherapy poses a risk for peripartum cardiomyopathy [19]. Since pregnancy may be an increased risk factor for developing cardiomyopathy in patients who have previously been exposed to anthracyclines and/or radiation, an assessment of the cardiovascular risk in these patients should be carried out before pregnancy or in the first few weeks of pregnancy [20].

### Radiation leading to cardiac side effects

Cardiac problems caused by thoracic radiation or following cancer therapy in childhood can manifest several decades later. Cardiovascular morbidity and mortality in childhood cancer survivors is mainly associated with anthracycline-treatment and radiation of the chest [21–24]. In this context it has to be emphasized that until now, systematic analyses only exist for those two treatments and long-term studies for all newer modalities are lacking. In computed tomography angiography, nearly 40% of childhood cancer survivors had coronary lesions more than 20 years after cancer treatment [25]. In this group of patients, development of cardiovascular risk factors should be closely monitored and stress testing (e.g. stress echocardiography, scintigraphy) even discussed in asymptomatic cancer survivors [26]. Typical chronic complications associated with radiotherapy include pericarditis, coronary artery disease, calcific valvular lesions, and myocardial fibrosis with mainly diastolic dysfunction [5, 27]. Chronic pericarditis is one of the most frequent radiation-induced cardiac side effects and also occurs following low-dose radiation [28]. Twenty percent of patients with chronic pericarditis develop a clinically relevant cardiac constriction. Among breast cancer patients who received radiation, coronary artery disease is the most frequent cardiovascular complication. Patients have an increased risk of developing coronary artery disease more than 20 years after radiotherapy [29, 30]. However, all patients should be advised to be aware of the potential for acute and chronic cardiac complications and report symptoms (e.g. fatigue, shortness of breath, edema) to their health care provider.

### Immune checkpoint inhibitors and cardiovascular side effects

Immune checkpoint inhibitors (ICI) are a novel class of drugs in systemic cancer treatment. ICI have been developed to restore the T cell-mediated immune response and improve the efficacy of anti-tumor treatments [31, 32]. Despite their excellent therapeutic effects, ICI may result in a broad spectrum of autoimmune-mediated side effects such as myocarditis [33, 18, 34–36]. In addition, the side effects can also manifest as arrhythmias (supraventricular and ventricular), conduction disorders with higher-grade blocks, but also acute myocardial ischemia, non-inflammatory cardiomyopathy, Tako-Tsubo syndrome, and pericarditis [37]. The mechanism of ICI-related cardiotoxicity is not yet fully understood. An autoimmune reaction against myocardial antigens is the assumed underlying pathomechanism in these patients. Autoantibodies against cTnT were observed in a patient with fatal Pembrolizumab-mediated myocarditis [38]. Bockstahler et al. proposed a potential mechanism where self-tolerance is abrogated by PD-1/PD-L1 blockade. Tissue damage releases self-antigens such as cardiac troponin from cardiomyocytes and antigen presentation by dendritic cells and inflammatory cytokines can lead to increased self-reactive CD4+ T-cells [39]. ICI-induced myocarditis occurs at a frequency of 1-2% and is associated with a relatively high mortality rate of 43-46%. Most cases of ICI-induced myocarditis occur in the first three months following the start of treatment [18] and more than 90% of patients show increased troponin levels [34]. Due to the potential severity of myocarditis, further diagnostics should be performed immediately if ICI-induced myocarditis is suspected, and ICI should be stopped. An ischemic aetiology or other underlying causes of the increase in troponin must be excluded. Recently, Mirabel et al. described a case of late-onset giant cell myocarditis due to enterovirus in a patient on long-term ICI therapy [40]. If myocarditis is confirmed (MRI or biopsy), glucocorticoids are considered as first line treatment (initially 1-2 mg/kg prednisolone, alternatively 1 g methylprednisone in non-responders) [41]. If corticosteroids are insufficient or if instability persists other immunosuppressants, including mycophenolate mofetil, tacrolimus, infliximab (note: infliximab is contraindicated at high doses in patients with heart failure [42]), antithymocyte globulin, or intravenous immunoglobulin and plasmapheresis can be used as second line options [43]. Recently, treatment with the CTLA-4 agonist abatacept has been shown to be successful in treating a patient with of severe, glucocorticoid-refractory ICI-induced myocarditis [44]. Abatacept is an inhibitor of T cell activation by blocking CD80 and CD86 and has originally been approved for the treatment of rheumatoid arthritis. A clinical study on using abatacept in the treatment of patients with ICI-associated myocarditis is currently being planned [43].

## Baseline risk assessment in cardiooncology

In the last years, several factors that significantly increase the risk for cardiotoxic effects in patients undergoing cancer therapy have been identified. Classical cardiovascular risk factors, preexisting cardiac disease, and previous cardiotoxic cancer treatment predispose to cardiovascular complications due to cancer therapy. Available data in this context is primarily based on anthracyclines and HER2-inhibitors and no strong data on other cardiotoxic drugs exists. The risk of developing cardiovascular side effects is generally higher in the younger (aged < 18 years) and older population (aged > 65 years) and further increases in the presence of two of the following conditions: diabetes mellitus, hyperlipidemia, arterial hypertension, obesity, and smoking [21, 45–49]. So those factors should be acknowledged prior to the initiation of a specific therapy and modifiable risk factors should be optimized (e.g. treatment of hypertension prior to starting VEGF inhibitors). Furthermore, radiation increases the risk of cardiotoxic effects of certain chemotherapeutic agents (e.g. anthracyclines)[50]. Exposure to high doses of radiation (> 30 Gy), anterior or left chest irradiation location, and the absence of shielding designate highest risk for radiation-induced heart disease [51].

Recently, the Heart Failure Association of the European Society of Cardiology together with the International Cardio-Oncology Society have emphasized the meaning of baseline risk assessment in cancer patients and proposed recommendations and a new risk assessment tool for patients planned to be treated with potentially cardiotoxic cancer therapies [52].

### Diagnostics and monitoring in cardiooncology

To monitor cardiovascular complications associated with cancer treatment, onco-cardiological assessment should include patient´s history, physical examination, electrocardiogram (ECG), echocardiography and biomarkers (e.g. troponins and natriuretic peptides). The ideal strategy for detection of cardiotoxicity is to compare measurements during cancer therapy with those obtained before initiation of the therapy. The identification of concomitant cardiovascular diseases is central in risk assessment. Patient´s history and physical examination can help to identify pre-existing heart failure and atherosclerotic manifestations.

### Detecting cardiotoxicity—the impact of imaging

Echocardiography plays a crucial role in the diagnosis of cardiotoxicity [53]. The current recommendations endorse the modified biplane Simpson´s technique by two-dimensional (2D) echocardiography as the standard method for left ventricular ejection fraction (LVEF) determination [11]. The drawback of this method lies in its reproducibility—the test-retest variability in LVEF measurement using 2D echocardiography is up to 10% [54]. For this reason, the sensitivity for the detection of small changes in LV function is low which weakens the strength of this method for serial measurements. Three-dimensional (3D) echocardiography offers a more precise way of measuring left ventricular volumes and therefore LVEF [55] and has been shown to have less variability in the measurements compared to the 2D methods [54]. Hence, 3D echocardiography should be used for serial monitoring of cardiac effects of chemotherapy whenever possible. The recommendations for chamber quantification from ASE and EAE established a LVEF ≥ 52% for men and 54% for women as a normal reference range [56]. A LVEF of 50-55% is borderline low and considered as a high risk for potential cardiotoxic effects of chemotherapy [57]. Deterioration of left ventricular ejection fraction (LVEF) appears to be a relatively late marker [58]. Calculation of global longitudinal strain (GLS) by two-dimensional speckle tracking echocardiography is more sensitive in the detection of subclinical LV dysfunction [59, 60]. GLS is a dimensionless measure of myocardial deformation that is analyzed by post-processing of apical images of the left ventricle using dedicated applications. A recent study has shown better interobserver agreement of GLS than for LVEF in patients undergoing potentially cardiotoxic chemotherapy [61]. A relative percentage reduction in GLS of > 15% is very likely to be abnormal [11]. Current guidelines recommend the comparison of GLS measured during chemotherapy cycles with baseline GLS values [11, 57]. Most of the data on the usefulness of GLS in predicting cardiotoxicity derive from therapies with anthracyclines or trastuzumab [62]. However, recently a study has shown the impact of changes in GLS also in the context of ICI-mediated myocarditis [63]. However, the gold standard of evaluation of left ventricular ejection fraction, volume and masses still remains cardiac magnetic resonance imaging (MRI). Cardiac MRI provides superior reproducibility of LVEF and the detection of small changes in LVEF and volumes compared to echocardiography [64], allowing tissue characterization of myocardial edema, inflammation and fibrosis. Its impact in the field of early detection of cardiotoxicity is increasing [65, 66]. In this context, novel MRI indices are currently being investigated and represent promising approaches for an earlier detection of cardiotoxicity in the future [67]. Nevertheless, the use cardiac MRI in clinical practice is limited by costs and limited availability.

### Biomarkers and cardiotoxicity

Several biomarkers (e.g. troponins, natriuretic peptides) have been studied for the detection of cardiotoxicity. Cardiac troponin levels are the gold standard biomarkers for the diagnosis of myocardial injury [68, 69]. In 2004 a study on troponin I in 703 breast cancer patients before and after chemotherapy showed that elevated troponin I levels were able to predict cardiovascular events. Further, patients with an early or persistent increase had a significant higher incidence of cardiac events (37% versus 84%) [70]. The same authors demonstrated that an increase in troponin I was a predictor of subsequent deterioration in LVEF in patients receiving high-dose chemotherapy [71]. However, another study in breast cancer patients failed to identify troponin I as early predictor of cardiac function [60]. Natriuretic peptides (e.g. N-terminal pro-BNP) reflect volume overload or increased wall stress. While increased BNP levels (> 100 pg/mL) were able to predict congestive heart failure in a population of 333 patients undergoing anthracycline therapy [72], two other studies showed no correlation between natriuretic peptide levels and LVEF deterioration [60, 73]. Reasons for inconsistent and conflicting results of studies using cardiac biomarkers include measurements in populations receiving different treatments, sample collections at varying time points, and the use of different cut-off values for “positive” biomarker values. However, in patients with elevated troponin more intensive monitoring may be recommended, while the ideal timing for troponin analysis and the cut-off for “positive” remain unclear.

Finally, microRNAs represent promising new approaches to predict cardiotoxicity. MicroRNAs such as miR-1 play an important role in the regulation of gene expression. Rigaud et al. demonstrated upregulated miR-1 levels in anthracycline treated breast cancer patients which were associated with future changes in LVEF ‘’[74].

### A glimpse of treating cardiotoxicity

The evidence for the treatment of cardiotoxic effects of cancer therapy is sparse. In 2652 patients treated with anthracyclines 226 patients developed cardiotoxicity (defined by a LVEF decrease of 10 percentage points to a value < 50%). Heart failure therapy including ACE inhibitors and/or beta-blockers was initiated early and 82% recovered from cardiotoxicity at least partially [13]. Based on this observational study in anthracycline therapy, the Working Group on Cardiooncology of the European Society of Cardiology recommends ACE inhibitors and beta-blockers in cancer patients with asymptomatic cardiac dysfunction to prevent the development of symptomatic heart failure or further dysfunction [5]. If LV function deteriorates during chemotherapy, the cardiooncology team has to balance between effective cancer therapy and cardiotoxicity. The recovery rate for trastuzumab-associated LV dysfunction is approximately 80% [75]. Thus, treatment should be suspended and re-evaluated after 3 weeks in the event of a reduction in the LVEF of 10% or more below 50%. This is broadly in line with the current position paper of the ESC, which, however, recommends the continuation of the therapy in the presence of ACE inhibitors if the LVEF drops to values between 45 and 49% [5]. Therapy should be suspended and reevaluated after 3 weeks in the event of reduction in the LVEF of 10% to more below 50% [76].

### Other cardiovascular side effects caused by cancer therapies

Beside myocardial dysfunction several cancer treatments predispose to cardiac arrhythmias [5]. Thus, an electrocardiogram (ECG) should be performed to identify ECG anomalies and cardiac arrhythmias. Some chemotherapeutic drugs (e.g. Tyrosine kinase inhibitors) may cause prolongation of the QT interval [5]. To avoid potentially life-threatening arrhythmias, frequency-corrected QT interval (QTc) should be assessed on a 12-lead ECG before initiation of cancer therapy and monitored under therapy. QTc intervals of > 500 ms or QTc prolongation of > 60 ms are at high risk for torsades de pointes tachyarrhythmias [77]. Atrial fibrillation (AF) and cancer go along with a state of hypercoagulability. In general, the risk of AF is increased in cancer patients. Cancer patients without a relevantly increased risk for bleeding (e.g. intracranial tumor, hematologic malignancies with coagulation defects, cancer therapy induced thrombocytopenia, severe metastatic hepatic disease etc.) should undergo the same considerations regarding anticoagulation as non-cancer patients [78]. Subgroup analyses of the direct oral anticoagulants (DOAC) have shown their safety and efficacy in cancer patients [79].

The risk for thromboembolic events is increased due to cancer itself but also as a consequence of chemotherapeutics and their administration routes affecting up to 20% of hospitalized patients [5]. For example, the combination of chemotherapy with VEGF inhibitors increase the risk of venous thromboembolism up to six-fold [80]. But also new therapeutic approaches as immunomodulatory drugs have been shown to significantly rise the thromboembolic risk [76]. Recent guidelines have incorporated DOACs into the actual treatment recommendations for venous thromboembolism – especially for patients with low bleeding risk who do not have gastrointestinal or genitourinary tract malignancies, severely depressed kidney function or relevant drug-drug interactions [81].

The ECG may also be indicative of myocardial ischemia. Mechanisms resulting in myocardial ischemia are manifold including vasospastic effects, endothelial injury, arterial occlusion, and defects in lipid metabolism. Typical ischemia-inducing drugs are Fluoropyrimidines (5-fluorouracil, capecitabine, gemcitabine), platinum compounds (cisplatin), and VEGF inhibitors (bevacizumab, sorafenib, sunitinib) [5]. If myocardial ischemia is suspected due to medical history and/or ECG, further work-up is important in those patients. But it must be acknowledged that the therapeutic options may be limited due to the restricted use of antiplatelet therapy in patients with (chemotherapy-induced) thrombocytopenia or increased bleeding risk. Platelet (PLT) number cut-offs for different antiplatelet regimens have been proposed (aspirin >10,000 PLT, aspirin+clopidogrel >30,000 PLT, ticagrelor and prasugrel > 50,000 PLT) [82]. Significantly reduced duration of dual antiplatelet therapy should be discussed in these cases. Close coordination between the oncologist and the cardiologist is crucial in these cases in an interdisciplinary team approach.

## Conclusion and perspective

Careful baseline cardiac assessment and monitoring are essential in all patients receiving a potentially cardiotoxic anti-cancer therapy and are best realized in close cooperation between oncologist and cardiologist. Patients should be advised to be aware of the potential for acute and long-term cardiotoxicity and report symptoms to their health care provider. A cardiology consultation should be considered if the LVEF is < 53%, GLS is below the limit of normal [83] and/or troponin levels are elevated. Female survivors of childhood cancer should undergo cardiological screening before pregnancy. In addition to cardiac dysfunction, which is certainly the most common and most serious manifestation of cardiotoxicity, the risk of other manifestations such as cardiac arrhythmias, myocardial ischemia, thrombotic events, but also—especially after a long period of time after radiation—the occurrence of pericarditis and valve diseases must not to be disregarded. Currently, diagnostic and treatment algorithms in cardio-oncology are largely based on expert consensus. Further research is needed to assess long-term benefits of cardiac surveillance and effects of cardioprotective therapy.

## Data Availability

Data sharing not applicable to this article as no datasets were generated or analysed during the current study.
